# Socio-Economic Factors Predicting Dental-Related Admissions in a Pediatric Population in the United States: Findings From the National Kids' Inpatient Database

**DOI:** 10.7759/cureus.105196

**Published:** 2026-03-13

**Authors:** Chintan Desai, Bedant Chakraborty, Jagdish Desai, Nilesh Dankhara, Sangeeta Gajendra

**Affiliations:** 1 Community Dentistry, Eastman Institute for Oral Health, University of Rochester, Rochester, USA; 2 Pediatrics/Neonatology, Pediatrix Medical Group, Austin, USA; 3 Community Dentistry, Eastman Institue for Oral Health, University of Rochester, Rochester, USA

**Keywords:** adolescent, child, dental caries, healthcare disparities, hospitalization, oral health, socioeconomic factors

## Abstract

Objective: To describe the trend of dental-related admissions from 2000 to 2019 in pediatric populations and to investigate socio-economic factors predicting dental-related admissions in this cohort.

Methods: The national multicentre Kids’ Inpatient Database (KID) Healthcare Cost and Utilization Project (HCUP) data were utilized from 2000 to 2019 for this study. Dental-related admissions were identified by scanning diagnosis fields DX1 through DX2 for International Classification of Diseases (ICD)-9-CM years and I10_DX1 through I10_DX40 for ICD-10-CM years. A binary outcome variable was created to flag any hospitalization with a dental-related diagnosis in any listed diagnosis position. While this approach maximizes capture of dental-related admissions, we acknowledge that inclusion of secondary diagnosis fields may introduce some degree of misclassification, as dental diagnoses in non-primary positions may not always represent the principal reason for hospitalization. This is noted as a study limitation. Statistical Analysis System (SAS) 9.4 was used for all the statistical analyses for this complex sample design.

Results: Among a total of 21,497,670 pediatric hospital admissions from 2000 to 2019, dental-related admissions were 2.7 cases per 1,000 pediatric admissions. The average admission rates were stable over the years, but an increasing trend (3.3% to 5.8%) was seen for the children aged 5-10 years. Among the top 10 causes of dental-related hospital admissions, periapical abscess was the most common, followed by maxillary hypoplasia. A statistically significant upward trend was found in the admission rate due to periapical abscesses and maxillary hypoplasia. In contrast, a declining trend was noted in jaw-cranial base diseases and maxillary hyperplasia anomalies.

Conclusion: Pediatric dental-related hospital admissions increased over time, driven by preventable conditions such as periapical abscesses. Differences in hospitalization risk by socio-demographic variables suggest that barriers to access timely preventive and specialty dental care may contribute to avoidable inpatient admissions.

## Introduction

Dental caries is one of the most common chronic conditions affecting young children, particularly in the primary (baby) teeth of those aged 2-5 years. While the overall prevalence of dental caries has declined since the 1970s, national data reveal persistent disparities across racial, ethnic, and socio-economic groups. According to the 2011-2016 National Health and Nutrition Examination Survey (NHANES), approximately 23% of children in the 2-5 year age group experienced caries in their primary teeth, with significantly higher rates observed among different race/ethnicity groups, such as Black, Mexican American, and children from low-income families [1]. According to the Centers for Disease Control and Prevention (CDC), the key findings from the NHANES data of 2015-2016 were that the prevalence of untreated caries in primary or permanent teeth among children aged 2-19 years was 13.0% [2]. The prevalence of untreated dental caries among children aged 2-5 was 8.8%, which was lower than it was among children aged 6-11 (15.3%) and 12-19 (13.4%) [2]. 

A study by Vasiredddy et al. reviewed the National Survey of Children's Health 2016 to 2019 data and reported that children, especially those who lack a stable medical home, originate from low-income families, lack insurance, and have a history of untreated dental caries, are at higher risk of developing dental caries and not receiving treatment [3]. Tooth decay can result in discomfort, infection, and tooth loss if not treated. It has been reported that loss of teeth may not only lead to medical issues but also affect psychologically, and be socially damaging, as well as result in functional limitations [4]. Dental caries among children has been associated with greater healthcare expenses, along with school absenteeism associated with emergency dental visits [5,6]. Moreover, dental diseases in children have also been significantly linked to lower parental workdays, apart from affecting school performance in children. As reported by Naavaal and Kelekar, school-age children wasted an estimated 142 million hours a year on average on dental visits, of which 34.4 million hours per annum are contributed by acute and unplanned dental visits [7].

A study conducted on 322 native Alaskan children revealed that the total annual costs for treatment of dental caries and whole mouth dental restorations averaged $258,000 and $1.5 million, respectively [8]. In addition, the burden on the state-run Medicaid program alone was reported to be between $100 to $400 million annually for the management of Early Childhood Caries (ECC) [9]. Recent evidence demonstrates that dental caries imposes a substantial and inequitable economic burden globally, with the highest per-person costs consistently concentrated among the most socio-economically deprived groups. Dunleavy et al. reported that uniform implementation of preventive strategies could significantly reduce direct costs for caries management, with the greatest reductions occurring within high-deprivation populations [10].

The medical and dental visits among children and adolescents aged 6-17 years have increased from 2003 to 2019 [11]. However, researchers reporting on pediatric non-traumatic dental condition (NTDC) visits in the emergency departments (EDs) in the United States (US) suggest that ED visits decreased over time following the implementation of the Affordable Care Act from 103.1 to 89.3 per 10,000 ED visits between 2010 and 2017 [12]. On the contrary, ED visits increased from 51% to 65.3% for children with Medicaid during the study period [12]. The researchers suggest that the factors associated with NTDC-related ED visits included age, primary payor, residence, household income, and comorbidity score. ​Despite the decrease in NTDC visits, children from low socio-economic backgrounds continue to rely on EDs for dental conditions at higher rates [12].

Dental diseases have been significantly linked to lower parental workdays, as well as have impacted school performance in children. The functional, psychological, and social facets of a child's well-being can be impacted by dental health. When children experience oral pain, it leads to numerous adverse outcomes, including poor learning, stunted growth, poor eating habits, decreased sleep, and behavioral issues. Inadequate dental health during development also affects the development of socialization and interaction, speech development, and self-esteem processes [13,14]. Thus, the present study was conducted to investigate the socio-economic factors predicting dental-related admissions using the Kids' Inpatient Database (KID) Healthcare Cost and Utilization Project (HCUP) from 2000 to 2012 and 2016 to 2019.

## Materials and methods

The study population included participants of the national multicenter KID HCUP from 2000 to 2012 and 2016 to 2019. The HCUP is a collection of longitudinal hospital care data in the US since 1988, collected by collaborative efforts of state entities, the federal government, and industry partners, with the sponsorship of the Agency for Healthcare Research and Quality (AHRQ) [15]. The data is gathered primarily from community hospitals across participating states, which include 48 states and the District of Columbia, representing 98% of the US population [15]. The database is constructed from hospital administrative data, i.e., billing records and various health services categories such as inpatient, outpatient, ED, and ambulatory services from hospital-owned facilities. Furthermore, the State Ambulatory Surgery and Services Database (SASD) not only includes data from ambulatory surgery and outpatient services from hospital-owned facilities but also includes data provided by facilities not owned by hospitals [16]. The HCUP does not incorporate services rendered at physician offices and lacks reliable information on pharmacy, laboratory, pathology, or radiology services [16].

The KID database, derived from the State Inpatient Database (SID), is one of the largest publicly available all-payer datasets for pediatric inpatient care in the US, capturing data on children and adolescents under the age of 21. When weighted, the KID estimates approximately 6 million pediatric hospitalizations, making it a robust resource for analyzing both common and rare health conditions [17]. 

For this study, children aged 1-15 years with dental-related diagnoses were identified using the 10th revision of the International Classification of Diseases Clinical Modification (ICD-10-CM) (e.g., K00 to K09) (Table 1) [18]. The primary outcome (dependent variable) was dental-related hospital admission, defined as a binary variable (1 = admission for a dental condition; 0 = no dental admission). The key predictor variables (independent variables) included social determinants of health such as gender, race/ethnicity, family income, insurance type, geographic region, and urban-rural location.

**Table 1:** ICD-10 CM codes ICD: International Classification of Diseases

Code	Description
K00	Disorders of tooth development and eruption
K01	Embedded and impacted teeth
K02	Dental caries
K03	Other diseases of hard tissues of teeth
K04	Diseases of pulp and periapical tissues
K05	Gingivitis and periodontal diseases
K06	Other disorders of gingiva and edentulous alveolar ridge
K08	Other disorders of teeth and supporting structures
K09	Cysts of oral region, not elsewhere classified

The present study comprised of children aged 0-15 years who were hospitalized in facilities that were part of the KID during the period ranging from 2000 to 2012 and 2016 to 2019. For this analysis, the de-identified 2019 KID dataset containing hospital discharge data from the AHRQ-HCUP website was used under appropriate licensing [17]. All analyses accounted for the complex sampling design of the KID database, including application of discharge weights and stratification variables, to ensure nationally representative estimates of pediatric dental-related hospitalizations.

Ethical considerations

This study was based on analysis of de-identified, publicly available data from the KID HCUP. As no identifiable private information was used, and no direct contact with human subjects occurred, institutional review board (IRB) approval was deemed exempt for this analysis.

Statistical analysis

To evaluate factors associated with dental-related hospital admissions, logistic regression models were employed. Demographic and socio-economic characteristics were entered as independent variables to assess their predictive influence. This study emphasized inferential statistics, reporting on odds ratio estimates to quantify associations and Type 3 Analysis of Effects to determine the statistical significance of each predictor. A significance level of p < 0.05 was used for all analyses. All statistical analyses were performed using Statistical Analysis System (SAS) version 9.4, accounting for the complex survey design of the KID database through appropriate use of sampling weights and stratification variables to generate nationally representative estimates.

## Results

**Figure 1:**
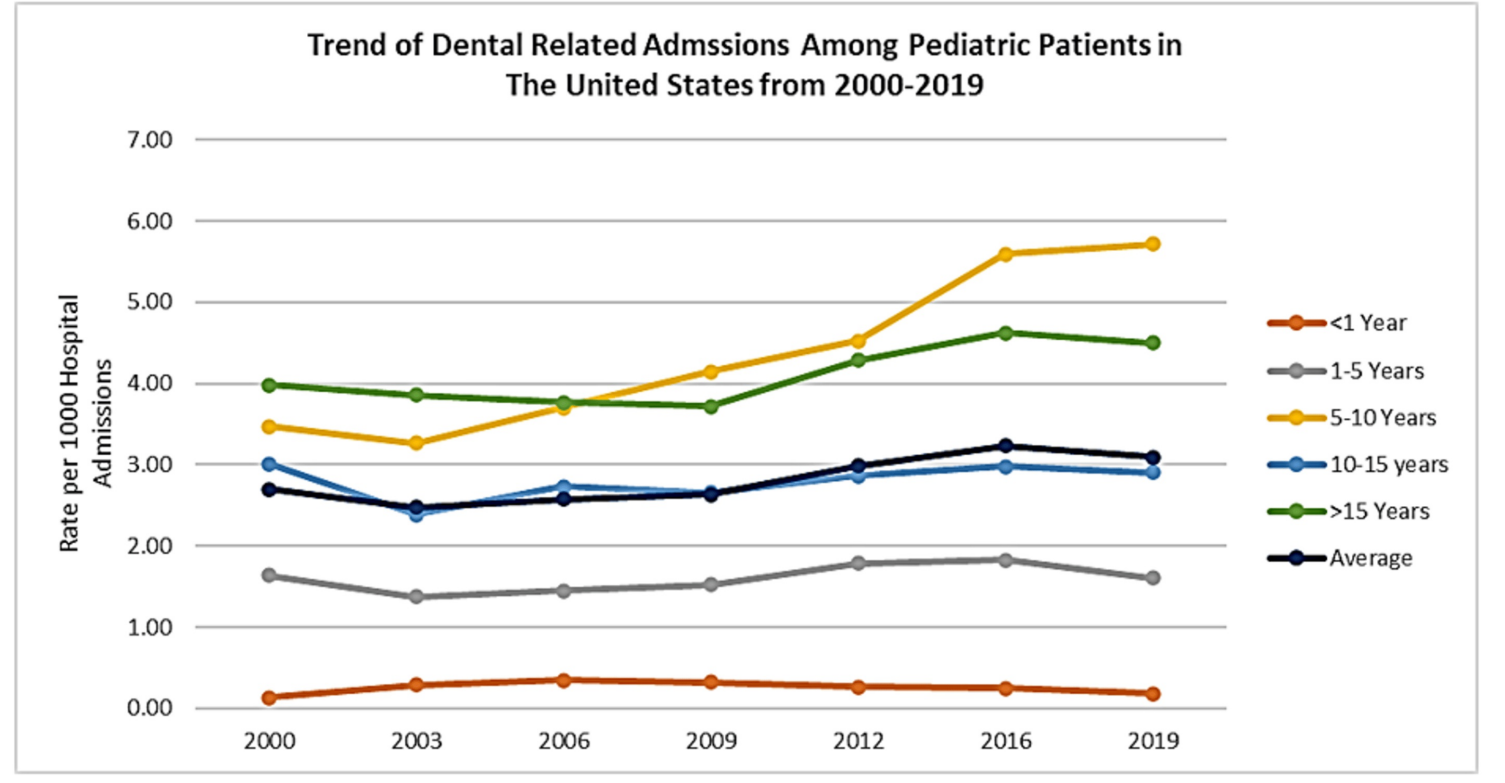
Dental admission trends over time

A total of 21,497,670 pediatric admissions from 2000 to 2019 were analysed. The overall rate of dental-related hospital admissions among children aged 0-15 years increased from 2.7 per 1,000 admissions in 2000 to 3.8 per 1,000 in 2019. Infants (0-1 year) consistently had the lowest admission rates (0.2 per 1,000), while the highest rates were observed in children aged 5 to 10 years, increasing markedly from 3.3 per 1,000 in 2013 to 5.8 per 1,000 in 2019 (Figure 1). Temporal trends in dental-related admission rates were examined descriptively using weighted estimates derived from PROC SURVEYFREQ across each available KID survey year. Survey weights (DISCWT), stratification variables (KID_STRATUM), and cluster variables (HOSP_KID) were applied in all analyses. No formal joinpoint or regression-based trend test was performed; trend descriptions are based on the direction and magnitude of changes in weighted point estimates across the study period.

**Figure 2:**
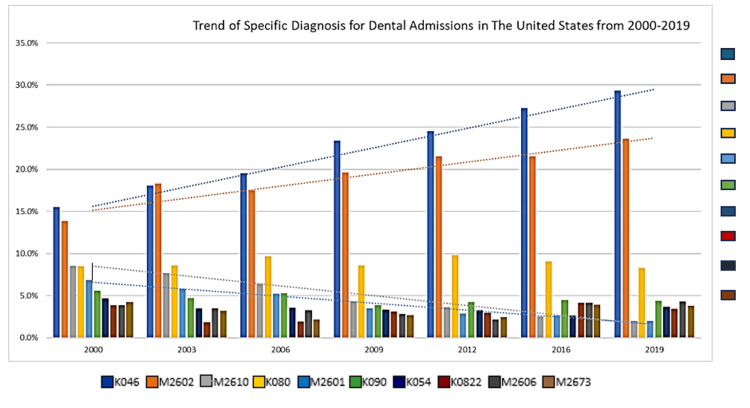
Specific Diagnosis for Dental Admissions from 2000-2019

**Figure 3:**
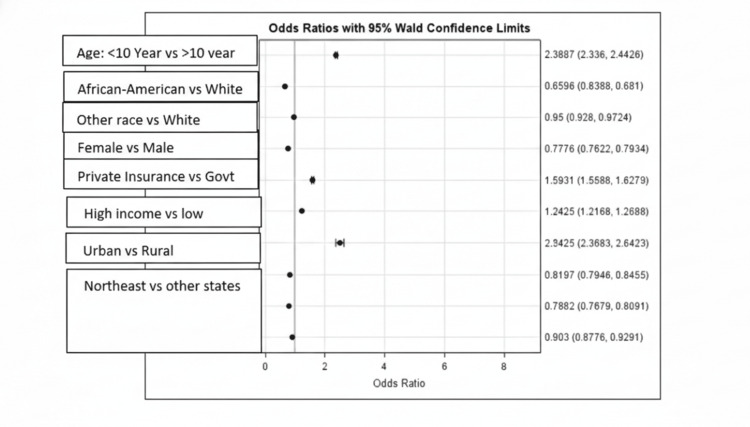
Socio-demographic analysis vs. dental-related admissions

Diagnostic patterns of dental-related hospitalizations over time are illustrated in Figure 2. The most common cause of dental-related admissions was periapical abscess (K046), followed by maxillary hypoplasia (M2602). Admissions due to both conditions showed a significant upward trend, whereas jaw-cranial base diseases (M2610) and maxillary hyperplasia (M2601) exhibited a declining pattern over time (Figure 2). Socio-demographic characteristics associated with dental-related hospital admissions are summarized in Figure 3. The analysis revealed that dental-related admissions were more likely among children aged 10 or older, males, White children, those with private insurance, and residents of urban areas, higher-income households, and northeastern states (Figure 3).

Multivariable logistic regression results examining predictors of dental-related hospital admissions are presented in Table 2. The regression analysis revealed several significant predictors of dental-related hospital admissions (Table 2). Children aged ≤10 years were notably less likely to be admitted for dental conditions than older children (adjusted odds ratio (AOR): 0.42; 95% confidence interval (CI): 0.41-0.43). African American children had lower odds of admission than White children (AOR: 0.66; 95% Cl: 0.64-0.68), and girls were less likely than boys to experience dental-related hospitalizations (AOR: 0.78; 95% Cl: 0.76-0.79). Insurance type also influenced admission rates, with children covered by government insurance showing reduced odds (AOR: 0.63; 95% Cl: 0.61-0.64) relative to those with private or self-pay coverage. Similarly, children from households with income below the 50th percentile had decreased likelihood of admission (AOR: 0.81; 95% Cl: 0.79-0.82). Rural residence was strongly associated with reduced odds of hospitalization for dental issues compared to urban settings (AOR: 0.40; 95% Cl: 0.38-0.42). Regional differences were also observed: children from the northeast had higher odds of admission (AOR: 1.12; 95% CI: 1.08-1.14) compared to those in the west, whereas those in the midwest (AOR: 0.91; 95% Cl: 0.88-0.94) and south (AOR: 0.87; 95% Cl: 0.85-0.90) had slightly lower odds (Table 2). These findings underscore the influence of demographic and socio-economic factors on pediatric dental hospitalization rates. All variables in the analysis of effects showed statistically significant associations with dental-related admissions (p <0.0001), reinforcing the importance of examining these predictors through regression rather than descriptive summaries alone (Table 3).

**Table 2:** Association of dental-related hospital admission with socio-demographic factors, KID 2000-2012 and 2016-2019 The odds ratio indicates the likelihood of the outcome occurring based on each predictor variable. Models adjusted for all variables shown in the table. Reference categories: age >10 years, male gender, White race, private insurance, income ≥50th percentile, urban location, western region. CI: Confidence interval; AOR: Adjusted odds ratio; KID: Kids’ Inpatient Database

Parameters/Characteristics	AOR	95% CI (Lower - Upper)
Age (≤10 years vs. >10 years)	0.42	0.41-0.43
African-American vs. White	0.66	0.64-0.68
Other vs. White	0.95	0.93-0.97
Female vs. Male	0.78	0.76-0.79
Government Insurance vs. Private/Self	0.63	0.61-0.64
Income (<50th percentile vs. >50th percentile)	0.81	0.79-0.82
Rural vs. Urban	0.40	0.38-0.42
Midwest vs. West	0.91	0.88-0.94
Northeast vs. West	1.11	1.08-1.14
South vs. West	0.87	0.85-0.90

**Table 3:** Type 3 analysis effects

Effect	df	Wald Chi-Square	p-value
Age	1	5860.65	<0.0001
Race	2	659.74	<0.0001
Gender	1	602.58	<0.0001
Insurance Type	1	1778.05	<0.0001
Income Level	1	413.38	<0.0001
Urban vs. Rural	1	1077.36	<0.0001
Hospital Region	3	351.93	<0.0001

## Discussion

This study analyzed more than two decades of nationally representative pediatric inpatient data, encompassing over 21 million hospital admissions, and identified a modest yet notable increase in dental-related hospitalizations among children aged 0-15 years from 2000 to 2019. Although the absolute rise in admission rates was slight, the consistent upward trend, particularly after 2013, suggests a growing burden of severe dental conditions, such as periapical abscesses, that require hospital-based care [19]. Given that most pediatric dental conditions are preventable and can be effectively managed in outpatient settings, these findings raise significant concerns about persistent gaps in early prevention, timely access to dental care, and service continuity.

The highest dental-related hospital admission rates were observed among children aged 5-10 years, with a marked increase in more recent years. This period corresponds to mixed dentition, heightened susceptibility to dental caries, increased exposure to dietary sugars, and continued reliance on caregiver-mediated oral hygiene practices [20]. The predominance of periapical abscesses as the leading cause of admission strongly suggests that untreated dental caries progressing to infection remains a primary driver of pediatric dental hospitalizations [19]. These findings likely reflect missed opportunities for early detection and preventive intervention rather than acute or unavoidable disease, underscoring gaps within routine dental care, school-based programs, and primary healthcare settings. In contrast, consistently low admission rates among infants are expected, given limited tooth eruption and shorter cumulative exposure to cariogenic risk factors.

Limited contemporary data exist regarding the influence of socio-economic status on pediatric dental-related hospital admissions. This study, therefore, represents one of the most comprehensive analyses of the socio-economic determinants of pediatric dental-related hospitalizations. Contrary to traditional assumptions, dental-related hospitalizations were more common among children who were white, privately insured, and residing in urban areas, even after adjustment [21]. Several explanations may account for this pattern. Privately insured children may have greater access to hospital-based specialty care, elective surgical admissions, or well-established referral pathways that disproportionately capture complex dental or craniofacial conditions. Similarly, children living in urban areas may have increased proximity to tertiary care, which could contribute to higher utilization of hospital-based dental services. It is important to note that hospitalization rates captured in the KID database reflect healthcare utilization patterns and access to inpatient dental services, rather than the actual prevalence or severity of dental disease in the community. Children from higher-income households with private insurance and those living in urban areas may have greater access to hospital-based specialty dental and craniofacial services, more formal referral networks, and higher rates of elective surgical admissions, all of which would increase their representation in inpatient records. Conversely, the lower odds of dental-related hospital admissions observed among rural children and those covered by public insurance likely reflect structural access barriers rather than a lower burden of unmet dental need in those populations. Additionally, residual confounding related to referral patterns should be considered. Children who are referred to tertiary care centers or academic medical settings, which tend to be concentrated in urban and higher-income areas, may be disproportionately captured in the KID data. This referral bias could partially explain the observed regional and socio-economic gradients in hospitalization rates and should be considered when interpreting these findings.

Consistent with prior literature, the lower odds of dental related hospital admissions among rural children may similarly reflect structural access limitations rather than a lower burden of disease [22]. Rural communities often face shortages of pediatric dental specialists and limited availability of hospital based oral health services, which may suppress inpatient admissions despite substantial unmet need [23]. Collectively, these findings emphasize that hospitalization rates do not necessarily equate to disease prevalence, but instead reflect complex interactions among access to care, referral systems, insurance structures, and care seeking behavior.

Diagnostic trends reveal two distinct but clinically meaningful pathways to hospitalization. The increasing admissions due to periapical abscesses highlight preventable infectious sequelae of untreated caries, which are associated with substantial healthcare costs and avoidable morbidity [19]. Conversely, the rising prevalence of admissions related to maxillary hypoplasia likely reflects the surgical management of craniofacial conditions, including cleft lip and palate, requiring hospital-based intervention rather than failures in preventive care [24]. Declining admissions for jaw-to-cranial base diseases and maxillary hyperplasia may indicate shifts toward outpatient management, specialized referral centers, or evolving admission thresholds. Together, these patterns suggest that pediatric dental hospitalizations arise from both preventable disease processes and structural or congenital conditions, emphasizing the need for early, targeted prevention alongside coordinated specialty care pathways.

The lower odds of dental-related hospital admissions observed among African-American children compared with White children likely reflect differences in access to inpatient dental and specialty care rather than actual differences in disease burden. A substantial body of literature shows that racial and ethnic minority children experience higher levels of untreated dental caries and unmet oral health needs, suggesting that reduced hospitalization rates may instead signal delayed diagnosis, under-referral, or limited access to specialty services. For instance, Northridge et al. have highlighted that people from modest/low-income backgrounds, or those belonging to minor ethnicities, uninsured groups, immigrants, and rural populations are more vulnerable to suboptimal access to oral healthcare [25]. Regional variation was also evident, with children in the northeast having higher odds of dental-related hospital admissions than those in other regions. This pattern likely reflects regional differences in healthcare infrastructure, availability of pediatric dental specialists and tertiary care centers, and variation in clinical thresholds for hospital admission.

Studies by the Institute of Medicine, Luo et al., and Thomson et al. also suggest that regular dental visits not only improve oral health but also benefit general health [26-29]. Recognizing this, the Healthy People 2030 identifies increased use of the oral health care system as a Leading Health Indicator, emphasizing the role of preventive dental care [30]. However, the lower odds of dental-related hospital admissions observed among minority children in this study suggest that persistent disparities in access and utilization remain, reinforcing that coverage alone does not ensure equitable use of preventive dental services.

From a public health perspective, these findings highlight the need for targeted strategies to improve access to preventive dental care that address the selected socio-demographic and geographic covariates examined in this study. Differences observed by age, race, insurance status, geographic region, and urban-rural residence suggest that disparities in access to preventive and specialty dental care continue to shape pediatric dental-related hospitalization patterns. Efforts to reduce preventable admissions should prioritize improving timely access to outpatient dental services and strengthening referral pathways for children at higher risk of severe dental disease, particularly school-aged children and those from populations experiencing structural barriers to care [23]. Addressing these access-related gaps may help reduce reliance on hospital-based dental care and mitigate persistent inequities in pediatric oral health outcomes.

Limitations

While this study offers extensive insights, several limitations should be acknowledged. First, the use of administrative data from the KID database may introduce coding errors or misclassification of diagnoses or outcomes. Additionally, scanning secondary diagnosis fields for dental codes may result in capturing admissions where a dental condition was incidental rather than the primary driver of hospitalization, potentially leading to over-counting of dental-related admissions, particularly in years where secondary fields are more extensively populated. Second, although our study timeframe was 2000-2019, the lack of data from 2013 to 2015, due to a coding shift in ICD, disrupted long-term trend continuity. The KID includes only inpatient data and excludes dental care delivered in outpatient, emergency, or community settings, potentially underestimating the overall burden. These broader contexts were discussed to frame our findings, but were not directly analyzed. Socio-economic status in this study was approximated using a ZIP code-level income quartile variable, which assigns patients to income quartiles based on the median household income of their residential ZIP code as reported in US Census data. This represents an ecological proxy and is subject to ecological misclassification, where the assigned income quartile may not accurately reflect the individual or household-level socio-economic circumstances of the patient. ZIP codes encompass populations with varying degrees of income heterogeneity, and this mismatch between area-level and individual-level data may attenuate or distort the observed associations between income and dental-related hospitalization. Future studies using individual-level socio-economic measures, such as insurance status combined with household income data from linked administrative records, would improve the precision of these estimates. Lastly, the cross-sectional design limits causal inference regarding the relationship between socio-demographic factors and hospitalizations. Future longitudinal studies are needed to more definitively explore these associations.

Future directions

Future research should incorporate longitudinal data to assess the long-term impact of healthcare reforms, including the Affordable Care Act, on pediatric dental health outcomes. Additionally, qualitative studies exploring parental decision-making, healthcare-seeking behaviors, and cultural perceptions of dental care would provide deeper insights into the causes of hospitalization disparities. Research focusing on the effectiveness of community-based interventions and school-based oral health programs could inform the development of targeted strategies to reduce preventable dental-related hospital admissions.

## Conclusions

This study identifies a modest but significant increase in pediatric dental-related hospital admissions, particularly among children aged 5-10 years, mainly driven by preventable conditions such as periapical abscesses. Hospitalization rate varied by selected socio-demographic and geographic factors, including age, race, insurance status, and place of residence. Lower odds of admission among children with public insurance and those living in rural areas likely reflect barriers to accessing preventive and specialty dental care rather than a lower burden of disease. These findings highlight the disparities and inadequate access to dental care among various subgroups in the population. There is a need for targeted strategies to improve access to preventive dental care for underserved pediatric populations and reduce avoidable dental-related hospitalizations.
